# SLC6 transporter oligomerization

**DOI:** 10.1111/jnc.15145

**Published:** 2020-08-28

**Authors:** Kumaresan Jayaraman, Anand K. Das, Dino Luethi, Dániel Szöllősi, Gerhard J. Schütz, Maarten E. A. Reith, Harald H. Sitte, Thomas Stockner

**Affiliations:** ^1^ Institute of Pharmacology Center for Physiology and Pharmacology Medical University of Vienna Vienna Austria; ^2^ Institute of Applied Physics Vienna University of Technology Vienna Austria; ^3^ Department of Psychiatry New York University School of Medicine New York City NY USA

**Keywords:** neurotransmitter transporter, oligomerization, PIP2, psychostimulant, Quaternary structure, transporter‐mediated efflux

## Abstract

Transporters of the solute carrier 6 (SLC6) family mediate the reuptake of neurotransmitters such as dopamine, norepinephrine, serotonin, GABA, and glycine. SLC6 family members are 12 transmembrane helix‐spanning proteins that operate using the transmembrane sodium gradient for transport. These transporters assume various quaternary arrangements ranging from monomers to complex stoichiometries with multiple subunits. Dopamine and serotonin transporter oligomerization has been implicated in trafficking of newly formed proteins from the endoplasmic reticulum to the plasma membrane with a pre‐fixed assembly. Once at the plasma membrane, oligomers are kept fixed in their quaternary assembly by interaction with phosphoinositides. While it remains unclear how oligomer formation precisely affects physiological transporter function, it has been shown that oligomerization supports the activity of release‐type psychostimulants. Most recently, single molecule microscopy experiments unveiled that the stoichiometry differs between individual members of the SLC6 family. The present overview summarizes our understanding of the influence of plasma membrane constituents on transporter oligomerization, describes the known interfaces between protomers and discusses open questions.

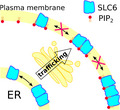

## Graphical abstract

We review the current knowledge on oligomerization of the solute carrier 6 (SLC6) family, which includes the transporters for the neurotransmitters dopamine, serotonin, and GABA. Early biochemical studies suggested dimer and tetramer configurations and identified several interaction motifs. Not all interfaces could be confirmed. Oligomers seem to become defined during transporter trafficking, are dependent on the plasma membrane PIP2 content, and affect substrate efflux. Simulations are starting to contribute to our understanding of transporter oligomerization, whereas recent single molecule experiments refined the oligomeric state by finding several non‐exchanging configurations for SERT, but only monomers and dimers for DAT.

**Abbreviations**
3M3FBS: 2,4,6‐Trimethyl‐*N*‐[3‐(trifluoromethyl)phenyl]benzenesulfonamide
AIM‐100: N‐[[(2S)‐oxolan‐2‐yl]methyl]‐5,6‐diphenylfuro[2,3‐d]pyrimidin‐4‐amine
BetP: betain transporter
CRAC/CARC: cholesterol‐binding motif
CRT: creatine transporter
DAFT: docking assay for transmembrane proteins
DAT: dopamine transporter
ECL: extracellular loop
ER: endoplasmatic reticulum
FILM: fluorescence lifetime imaging microscopy
FRET: förster resonance energy transfer
GABA: γ‐aminobutyric acid
GAT: γ–amino butyric acid transporters
GLYT: glycine transporters
hDAT: human dopamine transporter
hNET: human norepinephrine transporter
hSERT: human serotonin transporter
LeuT: small amino acid transporter
MDMA: 3,4‐methylenedioxymethamphetamine
NET: norepinephrine transporter
NSS: neurotransmitter:sodium symporter
PDZ: post‐synaptic density 95/Discs large/Zonula occludens‐1
PIP2: phosphatidylinositol‐4,5‐bisphosphate
PMF: potential of mean force
POPC: palmitoyl‐oleyl‐phosphatidyl‐choline
PROT: proline transporter
rDAT: rat dopamine transporter
SEC24: Protein transport protein SEC24
SERT: serotonin transporter
SLC6: solute carrier 6
TauT: taurine transporter
TMH: transmembrane helix
TOCCSL: thinning out clusters while conserving stoichiometry of labelling

## 1 INTRODUCTION

Neurons use electrical signals called action potentials to propagate information along axons and dendrites. Otto Loewi was awarded the Nobel Prize in Medicine in 1936 for the discovery that neurons use small signaling molecules (i.e., neurotransmitters) for communication between neurons. Upon neuronal excitation, neurotransmitters are released from the presynaptic neuron and bind to receptors on pre‐ and post‐synaptic neurons, eliciting new signals or modulating neuron activity. Normal neuronal function requires efficient removal of released neurotransmitters, which are typically achieved through re‐uptake by dedicated transporters. These transport processes are typically fast at synapses to allow for almost instantaneous response needed for synaptic transmission, whereas slower at other anatomic structures. Several diseases and disorders (e.g., Parkinson's disease, seizures, depression, schizophrenia, or attention deficit hyperactivity disorder) are associated with improper neurotransmitter clearance (Kristensen et al., 2011). Recreational drugs like cocaine, 3,4‐methylenedioxymethamphetamine (“ecstasy”) or methamphetamine interfere with transport and may lead to euphoric stimulation, addiction, and pathophysiological and psychological disturbances in the brain of drug users (Sitte & Freissmuth, 2015). The main neurotransmitters in the human brain are the excitatory neurotransmitters glutamate and acetylcholine, the inhibitory neurotransmitters γ‐aminobutyric acid (GABA) and glycine, and the neurotransmitters noradrenaline, serotonin, and dopamine, which are mostly involved in the regulation of neuronal activity. Neurotransmitters acting on the opioid and the cannabinoid receptors are involved in the regulation of pain sensation. Acetylcholine and noradrenaline are the key neurotransmitters in the vegetative neuronal system. The largest family of neurotransmitter transporters is the solute carrier 6 (SLC6) family, which achieves reuptake of released neurotransmitters by utilizing the electrochemical gradient of sodium. In humans, the SLC6 family includes transporters for the monoamines norepinephrine (NET), dopamine (DAT), and serotonin (SERT), glycine (GLYT), proline (PROT), γ–amino butyric acid (GAT), and taurine (TauT), as well as for creatine (CRT). Transport of cargo by secondary active transporters follows the alternating access model proposed by Jardetzky (Jardetzky, 1966). It posits that a transporter alternates between two conformational states, whereby access to the substrate‐binding site alternates between the intracellular side and extracellular side of the membrane.

**FIGURE 1:**
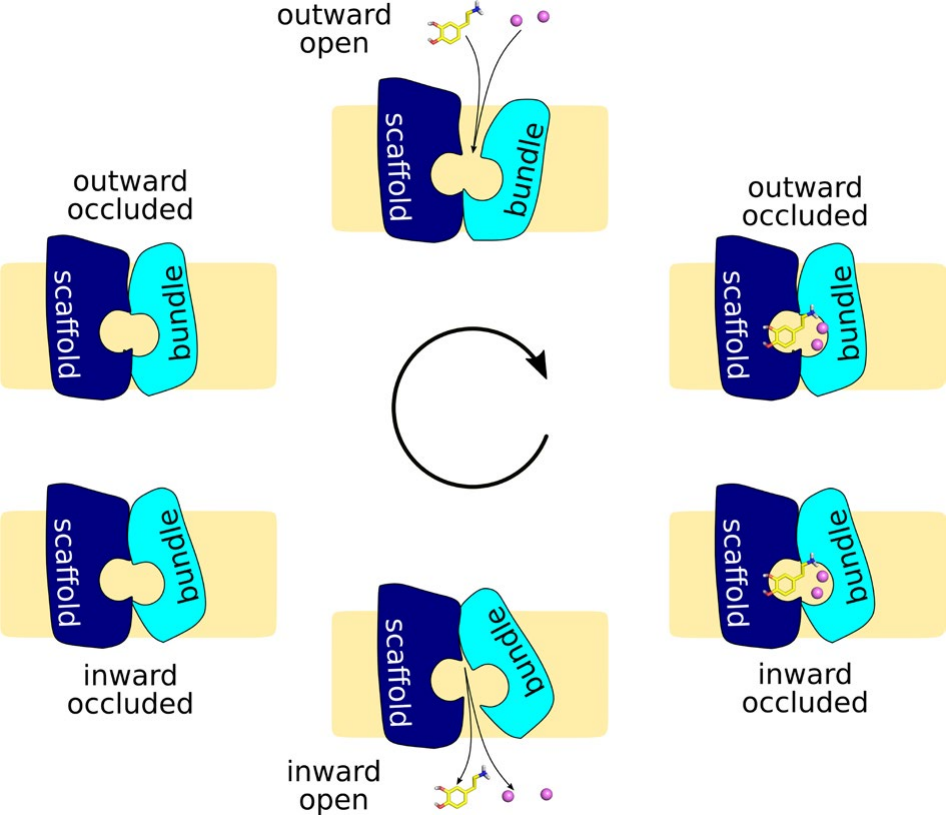
The sodium‐bound outward‐open conformation represents the resting state of SLC6 transporters. Upon substrate binding to the substrate‐binding site halfway through the membrane, the transporter occludes around the substrate, reaching first the outward‐occluded, then the inward‐occluded state. Substrate and co‐transported ions are then released to the cytosol from the inward‐open conformation. The transport cycle typically completes by returning empty to the outward‐facing state, with the exception of human SERT that carries a bound potassium ion

Crystal structures of the small amino acid transporter LeuT from *Aquifex aeolicus* unveiled the fold of the SLC6 protein family (Forrest & Rudnick, 2009). Importantly, these transporter structures revealed four conformations, which are seen as representing the outward‐open state (Singh, Piscitelli, Yamashita, & Gouaux, 2008), the outward‐occluded state (Yamashita, Singh, Kawate, Jin, & Gouaux, 2005), inward‐occluded (Gotfryd et al., 2020), and inward‐open state (Krishnamurthy & Gouaux, 2012) of the transport cycle. These 12 transmembrane helix transporters consist of two domains, the larger scaffold domain that anchors the protein in the membrane and the bundle domain that moves and rotates during the transport cycle (Figure 1), thereby alternating the access to the substrate‐binding site located midway through the membrane (Forrest & Rudnick, 2009).

The eukaryotic neurotransmitter transporter structures for dopamine from *Drosophila melanogaster* (Penmatsa, Wang, & Gouaux, 2013) and the human serotonin transporter (Coleman, Green, & Gouaux, 2016) confirmed a conserved fold from bacteria to humans and similarity of the main states of the transport cycle. However, the structures also revealed differences between LeuT and the eukaryotic transporters; most importantly, the position of the kinked transmembrane helix (TMH) 12 and the additional C‐terminal helix in the eukaryotic transporters. LeuT propagates as a stable dimer on SDS gels and has been crystallized as a dimer with TMH9 and TMH12 in the interface (Yamashita et al., 2005). The same dimerization interface does not exist in the eukaryotic transporters, because of the kink in TMH12 and the location of the C‐terminal helix protruding into the headgroup region of the membrane (Coleman et al., 2016).

## 2 EVIDENCE FOR OLIGOMERIZATION OF SLC6 TRANSPORTERS

Oligomerization of human monoamine transporters was recognized early (reviewed in (Sitte, Farhan, Javitch, & a, 2004; Sitte & Freissmuth, 2003). First evidence came from radiation inactivation studies, which detected oligomer formation that depended on inhibitor‐binding for human SERT (hSERT) in platelets (Mellerup, Plenge, & Nielsen, 1984), whereas finding monomers only in rat SERT (rSERT) (Plenge, Mellerup, & Nielsen, 1990). Using the same method, tetramers were detected for rat dopamine transporter (Berger, Farrell, Conant, Kempner, & Paul, 1994) and DAT from dogs (Milner, Béliveau, & Jarvis, 1994). Cross‐linking studies showed that rSERT forms variable degrees of dimers and tetramers (Jess, Betz, & Schloss, 1996). Using co‐immunoprecipitation, dimerization was shown in hSERT (Kilic & Rudnick, 2000), in hDAT (Torres et al., 2003), and hNET (Kocabas, Rudnick, & Kilic, 2003). Förster resonance energy‐transfer (FRET) measurements on hSERT (Bartholomäus et al., 2008; Fjorback et al., 2009; Just, Sitte, Schmid, & a, Freissmuth M., Kudlacek O., 2004), rGAT1 (Schmid, Just, & Sitte, 2001; Schmid, Scholze, et al., 2001; Soragna, Bossi, Giovannardi, Pisani, & Peres, 2005), and hDAT (Chen & Reith, 2008; Li, Cheng, Chen, & Reith, 2010; Sorkina, Doolen, Galperin, Zahniser, & Sorkin, 2003; Torres et al., 2003) confirmed the presence of oligomers in the plasma membrane, also in transfected neurons (Egaña et al., 2009). Using freeze‐fracture, hGAT1 expressed in Xenopus oocytes could be detected to form dimers (Gonzales et al., 2007). hSERT was also shown to form oligomers using fluorescence lifetime imaging microscopy (Fjorback et al., 2009). However, the exact stoichiometry of the oligomeric quaternary structure of SLC6 transporters remained unclear, because the wealth of data did not allow for recognizing a clear pattern as expected for such well‐defined structural arrangements. This, nevertheless, has been described in detail in ion channels, which show distinct trimeric, tetrameric, or pentameric structural arrangement (Marianayagam, Sunde, & Matthews, 2004).

## 3 STRUCTURAL MOTIFS IN DIMERIZATION INTERFACES

**FIGURE 2:**
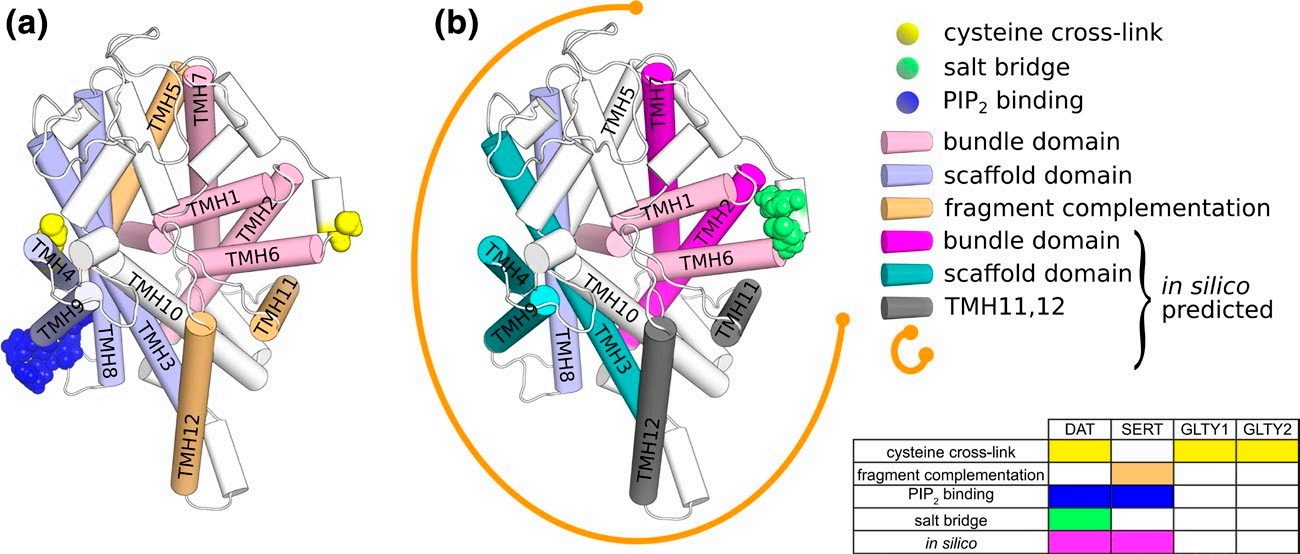
Experimentally and computationally determined oligomerization interfaces. (a) SLC6 transporter fold highlighting the experimentally identified oligomerization interfaces. (b) Oligomerization interfaces predicted by computational approaches. According to (Jayaraman *et at*, 2019) except the bundle domain, all transmembrane helices can contribute to oligomer interfaces (indicated as orange arc)

First indication of oligomerization interfaces (Figure 2) and involved residues came from cysteine cross‐linking experiments identifying C243 and C306 as residues within the hDAT dimer and tetramer interfaces (Hastrup, Karlin a, & Javitch J. a, 2001; Hastrup, Sen, & Javitch, 2003). Using the same approach and the equivalent position of hDAT C306, the glycine transporters GlyT1 and GlyT2 were also detected as dimers (Bartholomäus et al., 2008), confirmed by affinity purification and FRET. This was insofar surprising, as glycine transporters were believed to exist only as monomers at the plasma membrane (Horiuchi et al., 2001). A beta lactamase protein fragment complementation assay identified TMH11 and TMH12 to contribute to oligomeric interfaces in hSERT and also revealed indications for contributions by TMH5 and TMH6 (Just et al., 2004). Despite this big step forward, the specificity of dimer and tetramer arrangements remained difficult to reconcile with the scarce structural information available.

Starting from these initial results, known helix–helix interaction motifs were predicted and used to search for potential dimerization interfaces. The leucine zipper or leucine heptad repeat was frequently tested, as it is a well‐recognized helix–helix packing motif. It is a seven residue helical repeat motif, typically with leucines prominently exposed on one side of the helix. These leucines are packed in regular knobs‐into‐holes structures of parallel or anti‐parallel associated helices (William, Johnson, & Mcknight, 1988). Proposed leucine zipper dimer interfaces were tested for TMH2 by site‐directed mutagenesis and FRET in rGAT‐1 (Schmid, Just, et al., 2001; Scholze, Freissmuth, & Sitte, 2002) and hDAT (Sitte & Freissmuth, 2003; Torres et al., 2003). The studied mutations were found to affect folding, trafficking, and FRET, but the studies remained inconclusive as these changes could also stem from other effects than oligomerization deficiency. SLC6 structures (Penmatsa et al., 2013; Yamashita et al., 2005) revealed later that the postulated heptad repeat in TMH2 does not exist, because a π‐helix element in the middle of TMH2 change the direction of helical residue orientation. A π‐helix has five residues per helix turn instead of the four residues of classical α‐helices. Consequently, this π‐helix element in the middle of TMH2 leads to a 100‐degree rotational shift of following residue placements relative to the orientation expected for a classical α‐helix. The π‐helix element therefore disrupts the initially predicted heptad repeat in its center and places half of the leucine residues toward the core of the transporter. Clearly, the functional data for the rGAT‐1 and hDAT mutations in the heptad repeats of TMH2 have to be explained by transporter internal instead of oligomerization derived causes. The structures showed that a second proposed heptad repeat in TMH9 of hDAT is intact and surface exposed, but mutations of this motif did not affect transporter function (Torres et al., 2003), showing that the heptad motif in TMH9 does not contribute to transporter oligomerization.

The second prominent motif proposed in SLC6 transporter interfaces is the GXXXG helix crossing motif, in which two glycine residues are consecutively exposed on the same helix surface to allow for very close helix–helix association with defined helix crossing angles (Lemmon, Treutlein, Adams, Brünger, & Engelman, 1994). The identification of residue C306 to chemically cross‐link hDAT dimers in the presence of oxidizing reagents led to the proposal that the GXXXG motif in the middle of TMH6 could be part of a protein dimer interface (Hastrup et al., 2001). Structures of SLC6 transporters later showed that the GXXXG motif is part of the S1 substrate‐binding site in the center of the transporter and that it is involved in the flexing of TMH6 during substrate transport (Coleman et al., 2016; Krishnamurthy & Gouaux, 2012; Singh et al., 2008; Yamashita et al., 2005). Residue C581 in TMH12 of hDAT was shown to be a regulation site of transporter activity through palmitoylation (Vaughan & Foster, 2013), but it remains elusive if palmitoylation‐related effects would act through changes in transporter oligomerization.

## 4 TRAFFICKING AND OLIGOMERIZATION

Membrane proteins are synthesized by ribosomes attached to the ER membrane. After synthesis into the ER membrane, they traffic via the Golgi apparatus to their target compartment via a complex trafficking machinery (Jensen & Schekman, 2011). The monoamine transporters are recognized at the ER exit sites by SEC24C or SEC24D and loaded onto coat protein II (COP II) coated vesicles (Sitte et al., 2004). Recognition of the correctly folded transporters is contingent on the conformation of the C‐terminal helix; its deletion or mutation prevents surface expression (Chiu et al., 2005; El‐Kasaby et al., 2010; Koban et al., 2015; Moss et al., 2009; Sucic et al., 2011, 2013). A correctly formed interaction between the C terminus and the first intracellular loop is key for surface expression, as well as residues of the C‐terminal helix interfacing with the transmembrane part (Koban et al., 2015), suggesting that SEC24 might sense proper folding of individual transporters rather than oligomerization of folded transporters. Except for SERT, surface expression requires the presence of the post‐synaptic density 95/Discs large/Zonula occludens‐1‐binding domain at the C terminus (Bjerggaard et al., 2004; Rickhag et al., 2013; Torres et al., 2003).

Oligomerization was reported as important for trafficking for the SLC6 class of transporters (Sitte et al., 2004), but the proposed requirement of oligomer formation for SEC24 recognition and trafficking (Sitte et al., 2004) does not appear to be a stringent condition, as single molecule experiments have revealed that transporter monomers constitute the largest fraction of plasma membrane expressed hSERT and hDAT, amounting up to 40% of total surface expressed monoamine transporter (Anderluh et al., 2017; Das et al., 2019) However, in single molecule fluorescence microscopy experiments the observable distribution of co‐localized transporters may not be identical to the true oligomer count, since only active fluorophores can be counted and because the resolution of the experiment would not allow to distinguish true oligomer from oligomers connected by a scaffolding protein. Single molecule fluorescence microscopy experiments showed for hSERT that oligomer stoichiometry is defined before reaching the plasma membrane, as the oligomers become kinetically trapped by the high concentration of phosphatidylinositol‐4,5‐bisphosphate (PIP2), thereby fixing the stoichiometry (Anderluh et al., 2017). For hDAT, the subunit stoichiometry did not show PIP2‐dependence (Das et al., 2019), which appears to be a substantial difference between the two transporters.

## 5 SLC6 TRANSPORTER OLIGOMERS: COOPERATIVITY AND EFFLUX

Using two forms of SERT prepared with different epitope tags, Kilic and Rudnick found that dimeric forms of SERT displayed functional interactions between subunits (Kilic & Rudnick, 2000) and showed negative cooperativity. These findings have been reproduced by demonstrating that the hNET appears to be a functional homo‐oligomer (Kocabas et al., 2003). Cooperativity between transporters was also shown for hDAT (Zhen et al., 2015), using a conformationally locked mutant. By cross‐linking dimers, it was shown that one of the protomers is non‐functional at the time when the other one is actively engaged in transport (Zhen & Reith, 2018). Similarly, a Zn^2+^‐binding site was introduced in hDAT at the ECL3‐TMH6 border at position V310H (Norgaard‐Nielsen, Norregaard, Hastrup, Javitch, & Gether, 2002), which resulted in potent dopamine uptake inhibition upon Zn^2+^ binding (IC50 of 16 µM). The zinc‐dependent block of dopamine uptake could be a direct consequence of blocking transporter mechanics of DAT monomers, or of the cross‐linking of two transporters at the bundle domain, thereby blocking their movements.

A hSERT‐rGAT1 fusion concatemer‐based study revealed the functional interaction of oligomerized monoamine transporters, indicating that close transporter association is necessary for the action of amphetamine and congeners (Seidel et al., 2005; Sitte & Freissmuth, 2010). The study revealed that the presence of amphetamine analogs with substrate activity at hSERT triggered the release of GABA through rGAT1, which is inert to amphetamines. These data therefore indicate that transporter proximity is sufficient to couple transport activity between the normally non‐interacting hSERT and rGAT1. Direct transporter interaction was not required to induce efflux, indicating that (local) elevation of internal sodium might be the trigger. A high sodium concentration was later shown to be a prerequisite to trigger transporter‐mediated efflux (Mayer et al., 2016).

In addition to the impact that oligomerization plays for transporter‐mediated efflux, it was shown that psychostimulants such as methamphetamine and amphetamine influence transporter oligomerization. However, the extent and the underlying mechanisms remain largely unresolved. It was reported that multiple administrations of methamphetamine increase the formation of higher order oligomer of DAT in rats (Baucum, Rau, Riddle, Hanson, & Fleckenstein, 2004). This effect was associated with hyperthermia, while a single injection of methamphetamine or multiple injections of 3,4‐methylenedioxymethamphetamine failed to induce such an increase; this indicates that the observed formation of higher order DAT oligomers might be linked to persistent dopaminergic deficits (Baucum et al., 2004). However, other laboratories reported that amphetamines and the physiological substrate dopamine disrupt oligomer formation of hDAT (Chen & Reith, 2008; Li et al., 2010), whereas the action of the blocker cocaine had no effect on oligomerization when measured by co‐immunoprecipitation, and even increased cross‐linking with Cu^2+^ (Chen & Reith, 2008). These results also link oligomerization of DAT to endocytic or other modulatory mechanisms (Chen & Reith, 2008). Furthermore, cocaine‐induced formation of DAT oligomers was discussed as potential mechanism leading to cocaine tolerance, which can be reversed by treatment with amphetamine, a substrate that was proposed to disperse DAT oligomers (Siciliano et al., 2018).

## 6 DYNAMIC OLIGOMER STRUCTURES AND THE ROLE OF PHOSPHOINOSITIDES

PIP2 constitutes 1% of the membrane lipids and is only present in the intracellular leaflet of the plasma membrane. Via the PIP2‐Ca^2+^ signaling system, PIP2 is an important regulator for cell activation in response to various extracellular stimuli (Chang & Liou, 2016). Important second messengers are derived from PIP2 by lipolysis, including diacylglycerol, phosphateinositol‐(1,4,5)‐triphosphate (Czech, 2000; Kadamur & Ross, 2013), phosphatidic acid (Raben & Barber, 2017), and arachidonic acid (Bazinet & Layé, 2014). PIP2 is also known for its role in regulating membrane proteins (Czech, 2000; Suh & Hille, 2008), especially ion channels (Hansen, Tao, & MacKinnon, 2011; Hille, Dickson, Kruse, Vivas, & Suh, 2015; McLaughlin & Murray, 2005; Schulze, Krauter, Fritzenschaft, Soom, & Baukrowitz, 2003; Soom et al., 2001) but also transporters (Buchmayer et al., 2013; Hamilton et al., 2014), receptors (Yen et al., 2018), or proteins involved in vesicle exocytosis (Martin, 2015).

**FIGURE 3:**
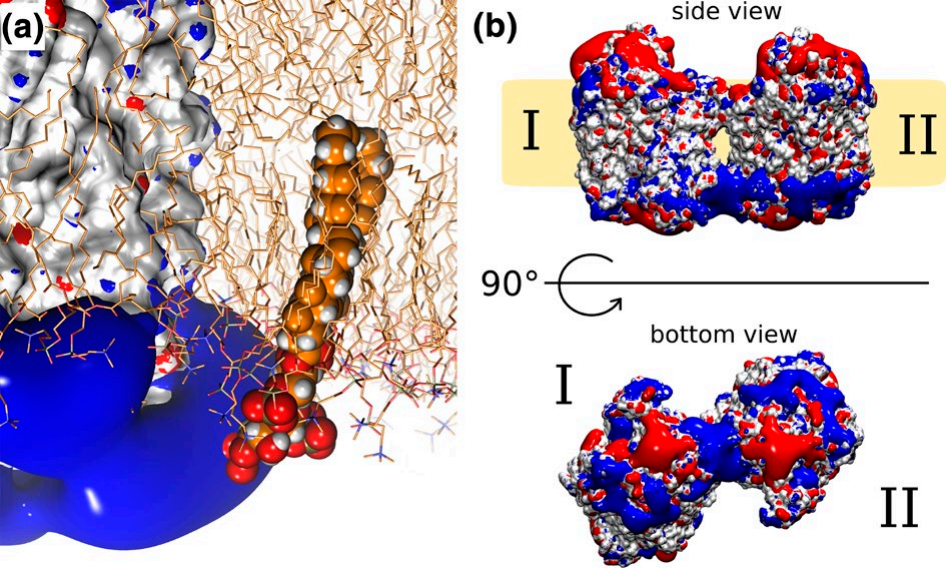
Electrostatic fields generated by monoamine transporters: (a) The positive electrostatic field in close proximity to intracellular loop 4 of hSERT is shown in blue rendering. A PIP2 molecule is modeled into the membrane for visualization purposes. The fourfold negatively charged headgroup of PIP2 can interact with the positive electrostatic field of hSERT while remaining membrane embedded. hSERT is shown in white surface rendering, the membrane as sticks. Standard coloring (negative potential in red, positive potential in blue) is used for the electrostatic fields. Image reproduced from Anderluh et al., 2017. (b) The electrostatic fields (shown as red and blue surfaces) of hDAT extends far into the plasma membrane, if two positively charged regions on the hDAT surface are juxtaposed, representing attractive fields for PIP2 binding. Image reproduced from Jayaraman et al., 2018

Direct binding of PIP2 to hSERT (Buchmayer et al., 2013) and hDAT (Hamilton et al., 2014) was shown by immunoprecipitation. Binding of PIP2 to monoamine transporters was additionally inferred from in vitro and in vivo evidence of changes in transporter activity by manipulation of PIP2 levels and transporter mutagenesis (Buchmayer et al., 2013; Hamilton et al., 2014). Modulation of PIP2 levels changed the activity of transporters, including substrate uptake and efflux as well as transporter‐mediated currents. PIP2 interacts with the transmembrane core of hDAT and hSERT (Figure 3; (Buchmayer et al., 2013) & (Belovich et al., 2019)). In addition, PIP2 regulates efflux through electrostatic interactions with positively charged residues of the distal N terminus of hDAT without affecting substrate uptake (Hamilton et al., 2014).

PIP2 was shown to interact with positively charged residues of hSERT present at the lipid–membrane interface (Buchmayer et al., 2013) (Figure 3a), and to stabilize protein dimers by contemporaneously interacting with lysines and arginines juxtaposed on both protomers (Jayaraman et al., 2018). Mutation of residues K352 and K460 on hSERT identified these residues as interaction hotspots (Buchmayer et al., 2013). More recently and similar to the above‐described findings for hSERT, PIP2 was shown to interact with intracellular loop 4 of hDAT (Belovich et al., 2019). In agreement with these experimental data, simulations employing hDAT have indicated that PIP2 strongly interacts with lysine and arginine residues exposed to the intracellular leaflet (Hamilton et al., 2014; Jayaraman et al., 2018; Khelashvili et al., 2015a, 2015b).

## 7 SINGLE MOLECULE MICROSCOPY ENLIGHTENS THE OLIGOMERIC DISTRIBUTION

**FIGURE 4:**
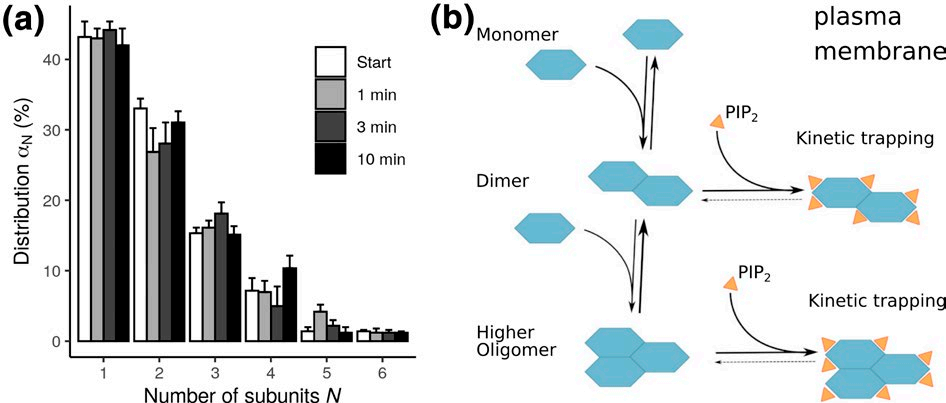
Transporter oligomerization: (a) The distribution of oligomeric size of hSERT follows an exponential decay as measured by single molecule imaging. (B) Model of transporter oligomerization and kinetic trapping by PIP2: SLC6 transporters are in a dynamic equilibrium of monomers, dimers, and higher oligomers in the case of hSERT. The occurrence of higher order oligomers decreases with higher oligomeric size. Oligomers are kinetically trapped by PIP2 interactions, which is a consequence of the orders of magnitude faster diffusion of PIP2, therefore effectively stabilizing existing oligomers by fast re‐binding

Besides regulating the function of individual transporters by direct interactions, it became evident that PIP2 plays an important role in transporter oligomerization. By single molecule imaging, we found that PIP2 kinetically traps hSERT in the plasma membrane and fixes its oligomeric state (Anderluh et al., 2017). These observations have been made possible by employing a methodology to thin out clusters in cells without altering the stoichiometry of the label thinning out clusters while conserving stoichiometry of labelling. In conjunction with single molecule brightness analysis, thinning out clusters while conserving stoichiometry of labelling allows for the quantification of the oligomeric state of mobile membrane constituents even under high expression levels: by photobleaching a small area of the plasma membrane all fluorophores within this area are switched off, whereas the fluorophores in the remaining regions of the plasma membrane remain active. Thereby, oligomers become either dark or retain their brightness. Brownian motion leads to recovery of the fluorescence signal; at the onset of this recovery process, the individual entities can be resolved as well‐separated diffraction‐limited signals. The brightness of each signal can be quantified and, upon comparison to the brightness of a single dye molecule, the oligomeric state can be inferred. Single molecule studies of hSERT (Anderluh et al., 2017; Anderluh, Klotzsch, Reismann, et al., 2014; Anderluh, Klotzsch, Ries, et al., 2014) and hDAT (Das et al., 2019) showed that the monoamine transporters do not form a specific quaternary structural assembly, as no specific oligomeric stoichiometry could be measured. Instead, in both the ER membrane and the plasma membrane a mixture of monomers up to at least pentamers was detected for hSERT (Figure 4a). The occurrence of higher order oligomers decreased with increasing oligomeric size, following an exponential decay. In contrast to hSERT, the distribution of hDAT was dominated by monomers and dimers. However, as aforementioned, the visible distribution may deviate from the true distribution. Furthermore, it remains unclear if higher order hDAT oligomers could be detected at an expression level comparable to hSERT, as hDAT showed 1–2 orders of magnitude lower surface expression. Of note, however, experimental evidence suggests that there is no such density dependence of the oligomeric state (Anderluh et al., 2017; Anderluh, Klotzsch, Reismann, et al., 2014; Anderluh, Klotzsch, Ries, et al., 2014; Das et al., 2019). Consistently, neither hDAT nor hSERT showed detectable subunit exchange over time scales of several minutes (Figure 4b). In contrast to the situation at the plasma membrane, at the ER level a continuous exchange of hSERT oligomer subunits takes place. Of note, the ER and plasma membrane differ considerably in their lipid composition; for example, the ER membrane reaches a rather low cholesterol level of 5% and is devoid of PIP2. Strikingly, subunit exchange rates of hSERT at the plasma membrane were similar to the ER membrane after PIP2 depletion.

The size of the hSERT oligomers was independent of transporter expression levels, which are hard to reconcile with the measured exponential decay of oligomer stoichiometry. After PIP2 depletion with continuous protomer exchange, the mean oligomeric size at the plasma membrane became indistinguishable from the mean oligomeric size in the ER at the same transporter densities. Thus, oligomerization of monoamine transporters is surprisingly insensitive to the lipid–membrane composition (Anderluh, Klotzsch, Ries, et al., 2014), except for the signaling lipid PIP2 (Anderluh et al., 2017). PIP2 kinetically traps the oligomers of hSERT at the plasma membrane by preventing protomer dissociation and further association with larger structures (Figure 4b).

The high concentration and the much faster diffusion of PIP2 relative to the monoamine transporters effectively fix the oligomeric state by swift PIP2 re‐binding before slowly diffusing transporters could separate. This rim of positively charged residues can create a large positive electrostatic field, which extends far away from the protein surface when mirrored by a second transporter in close proximity (Jayaraman et al., 2018). These positive electrostatic fields attract the negatively charged PIP2, acting as primary interaction site for PIP2 (Figure 3b). In accordance, simulations using a single hDAT (Hamilton et al., 2014) (Khelashvili et al., 2015a, 2015b) showed stable interactions of PIP2 with positively charged residues at the intracellular membrane–protein interface.

With the exception of PIP2, membrane lipids might generally play a minor role in neurotransmitter:sodium symporter (NSS) oligomerization. It was shown for hSERT (Anderluh et al., 2017; Anderluh, Klotzsch, Reismann, et al., 2014; Anderluh, Klotzsch, Ries, et al., 2014) that oligomer distribution and protomer exchange kinetics were indistinguishable between the ER membrane and the PIP2 depleted plasma membrane. Also, depletion of cholesterol did not affect oligomer size or stability of hSERT (Anderluh et al., 2017; Anderluh, Klotzsch, Reismann, et al., 2014; Anderluh, Klotzsch, Ries, et al., 2014) and hDAT (Das et al., 2019). Simulation studies investigating cholesterol interactions with hDAT (Zeppelin, Ladefoged, Sinning, Periole, & Schiøtt, 2018) and hSERT (Laursen et al., 2018) found binding sites that overlapped with the sterols observed in the crystal structure of dDAT and hSERT. Furthermore, it was found that cholesterol interacts with other transmembrane helices and binds to some of the CRAC/CARC motifs. In contrast to oligomerization, substrate uptake is strongly dependent on cholesterol in the plasma membrane (Jones, Zhen, & Reith, 2012; Scanlon, Williams, & Schloss, 2001). These data therefore imply that the membrane composition shows different and possibly independent roles for transporter oligomerization and for substrate transport.

## 8 IN SILICO STUDIES ON NSS TRANSPORTER OLIGOMERIZATION

Structures of the transmembrane domains of LeuT, hSERT and dDAT are now available. LeuT has been solved in four conformations, mostly in the presence of conformation stabilizing ligands and/or antibodies. Structures of hSERT without N‐terminus and the C‐terminus were solved in three conformations with the help of conformation stabilizing mutations, antibodies, and in the presence of ligands, whereas only one conformation is available for dDAT, which was solved in the presence of several ligands. A significant part of ECL2 of dDAT was removed to allow for transporter crystallization. These structures are assumed to represent snapshots along the path of the transport cycle, and therefore allow for using modeling and simulation techniques to investigate transporter structures, dynamics, and oligomerization. Some early simulations of NSS dimers were modeled based on the LeuT dimer interface (Brinkø et al., 2016; Koldsø, Autzen, Grouleff, & Schiøtt, 2013; Koldsø, Christiansen, Sinning, & Schiøtt, 2013; Koldsø et al., 2011). However, this interface cannot be formed in eukaryotic NSS transporters because of re‐positioning of TMH12 and the conserved kink in the center of TMH12. A LeuT‐based dimer leads to a rotation of the monoamine transporters relative to the membrane plane and therefore results in a hydrophobic mismatch, which affects the conformation of the functionally important bundle domain.

Gur et al. (Gur, Cheng, Zomot, & Bahar, 2017) created a dimeric model of hDAT using protein docking and found that a pair of salt bridges (R304 and E307) stabilized the hDAT dimer in a conformation that keeps the two C306 cysteines at cross‐linking distance. Site‐directed mutagenesis and chemical cross‐linking were used to confirm the importance of residues R304 and E307 for stabilizing the hDAT dimer in a cross‐linkable conformation. Interaction of the cell‐permeable furopyrimidine AIM‐100 was reported to trigger hDAT trimerization and to lead to dynamin, cholesterol‐rich microdomains, and actin cytoskeleton‐independent endocytosis (Cheng et al., 2019; Sorkina, Ma, Larsen, Watkins, & Sorkin, 2018). Three diverging models of the hDAT trimers were proposed by protein modeling and simulation (Cheng et al., 2019); two models were built using docking of hDAT monomers, the third was based on the BetP trimer (Ressl, Terwisscha Van Scheltinga, Vonrhein, Ott, & Ziegler, 2009), which has a topology diverging from LeuT, hSERT, and hDAT. However, the study remained inconclusive in terms of which of the three models would be stabilized by AIM‐100, as all three models were not fully consistent with all available data.

**FIGURE 5:**
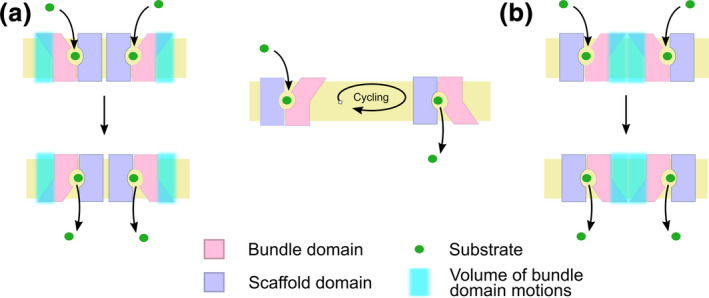
Motions of the bundle domain are important for effective transport. The bundle domain rotates during the transport cycle by switching between the inward and the outward facing state; this leads to lateral bundle movements of retraction and extension (cyan area). (a) The bundle domain can freely move during the transport cycle unless it interacts with other protomers. (b) Interactions that include the bundle domain need to be overcome. Additional energy is required for displacement of the adjacent protomer or for breaking of existing interactions. This effectively slows down the rate of substrate transport

Unbiased simulations starting from fully separated transporters require much higher computer power, but do not suffer from shortcomings stemming from human judgment, the procedure of oligomer model building, or from the limited precision of docking programs. Oligomerization of hSERT (Periole, Zeppelin, & Schiøtt, 2018) was studied by long unbiased simulations, using a coarse‐grained representation of 16 or 64 hSERT molecules equidistantly inserted into a palmitoyl‐oleyl‐phosphatidyl‐choline membrane. The simulations showed extensive hSERT transporter aggregation, forming major clusters. This deviation from the experimentally observed cluster size of five members or smaller might be attributed to the too strong interactions between membrane proteins when using the applied Martini 2.2 force field (Javanainen, Martinez‐Seara, & Vattulainen, 2017). This force field dependent effect was strong, because of the high transporter density and the very long simulation times (30 or 250 μs) of the study. The observed interfaces were grouped into four clusters, revealing four dominant interfaces, two symmetric interfaces involving TMH3/4 or TMH12 and two asymmetry interfaces including TMH7‐TMH12 or TMH4/9‐THM2/11 or the respective protomers. The symmetric dimers were shown to be stabilized by strong interactions, using potential of mean force calculations. The asymmetric dimer interfaces included one helix of the bundle domain and were barely stabilized by attractive forces. In contrast to experimental data, the stability of the strongest symmetric dimer involving TMH12 was weakened by the addition of 30% cholesterol or 10% PIP2, added to both membrane leaflets. A large‐scale simulation approach investigating hDAT dimer formation using coarse‐grained Martini 2.2 force field employed the docking assay for transmembrane proteins approach (Wassenaar et al., 2015), using 512 independent simulations (Jayaraman et al., 2018) starting from a random distribution of relative transporter orientation. This setup suffers much less from the over‐stabilization of membrane proteins (Javanainen et al., 2017), because it uses only two proteins per simulation and employs a much larger number of much shorter trajectories. This comprehensive dataset revealed that hDAT can form several dimer configurations, which are clustered into four symmetric and four asymmetric geometries of varying likeliness. Helices found in the dimer interfaces were distributed over most of the transporter surface, but spared the bundle domain. Exclusion of the bundle domain was verified by potential of mean force calculations, showing that any transient contact observed during the unbiased simulations was not stabilized by attractive forces. Exclusion of the bundle domain from dimer interfaces would be advantageous for substrate transport, because it would avoid a potential energy barrier stemming from the large‐scale rotation of the bundle domain that leads to extensive lateral movements within the membrane (see Figure 5). Consequently, strong interactions would slow down the transport cycle because of the need to break transporter–transporter interactions or requiring lateral translation of transporter dimers.

## 9 CONCLUSIONS AND OPEN QUESTIONS

There is ample experimental evidence that SLC6 family members form oligomeric quaternary complexes at organellar membranes before reaching the plasma membrane. For the human SLC6 transporter family members SERT and DAT we know that once formed, transporter stoichiometry is fixed and protomers do not exchange at the plasma membrane on the timescale of minutes accessible by the applied methods. However, it remains unclear why the closely related transporters hSERT and hDAT differ in the stoichiometries of their quaternary arrangements (i.e., hSERT molecules can be found from monomers to a number of different oligomers, whereas hDAT is almost exclusively present as monomeric or dimeric species). The impact by membrane constituents may play a role; however, this has not yet been fully elucidated. The difference between hSERT and hDAT function with respect to PIP2 interactions has so far been attributed to either an interaction of PIP2 with the N‐terminal domain (hDAT) or a direct interaction of PIP2 with the core protein (hSERT). Most recently, an interaction of PIP2 with the core protein of hDAT has been described as well. It would be of relevance to assess whether interactions of PIP2 with the N‐terminal domain of hSERT would also have functional importance. However, it must be noted that this does not imply whether the N‐terminal domains play any role in the quaternary arrangement, at least this has not been suggested by any published evidence so far. Nevertheless, this conjecture would certainly require experimental verification.

Another open question is the existence of dimeric or multimeric species in the native environment of the monoamine transporters, that is, in live neurons. To date, we have only evidence from biochemical data (see above) and from transfected hippocampal neurons (Egaña et al., 2009) that transporters may exist as oligomeric complexes in native environments. So far, we are still lacking evidence from direct approaches such as single molecule microscopy, but such quantification would be important to correlate the in vitro data to neurons. The functional approaches remain too ambiguous in their interpretation and can thus not help to address this question, at least not the evidence gathered so far. Some indirect evidence, however, has been gathered from experiments on hippocampal slices where hSERT‐mediated efflux of preloaded tritiated transporter substrate was significantly reduced by the phospholipase C‐activating drug m‐3M3FBS (Buchmayer et al., 2013), the same substance which was later shown to reduce hSERT oligomers in vitro in single molecule microscopy experiments (Anderluh et al., 2017). Interestingly, a similar procedure to reduce PIP2 was shown to change locomotion in a behavioral assay in Drosophila melanogaster (Hamilton et al., 2014). Furthermore, observations showing that PIP2 hydrolysis increases subunit exchange poses the question whether physiological changes of PIP2 levels would alter oligomer formation and sizes at the high transporter concentrations found in synapses. Recently, it was reported that the small molecule AIM‐100 drives oligomer formation of DAT (Sorkina et al., 2018). Even though it is debated how this small molecule might act at hDAT (Wu et al., 2017), the initial results obtained biochemically and by FRET microscopy (Sorkina et al., 2018) received support by computational approaches (Cheng et al., 2019). Hence, it would be highly relevant to further investigate AIM‐100‐mediated induction of oligomer formation and internalization, and to unravel whether such changes would in turn be accompanied by changes in transporter functions.

Last but not least, one of the most crucial open questions is the physiological role of oligomer formation. Earlier hypotheses linked oligomer‐formation to trafficking and substrate‐triggered transporter‐mediated efflux (Seidel et al., 2005). Even though transporter substrates such as ephedrine or cathinone are naturally occurring alkaloids, it remains questionable whether response to such substances demonstrates a physiological role of transporter oligomerization, linking drug substrate uptake with dopamine efflux. Nevertheless, in the case of both dopamine and GABA transporters, reverse transport is known to be physiologically relevant (Falkenburger, Barstow, & Mintz, 2001) and as those for dopamine, the transporters for GABA are present as oligomers in the plasma membrane (Schmid, Scholze, et al., 2001). We currently do not understand enough about the transport process and whether it strictly follows “enzyme”‐like kinetics in both directions (Hasenhuetl, Bhat, Freissmuth, & Sandtner, 2019; Hasenhuetl et al., 2018); however, it may be a more complex, asymmetric process (Sitte & Freissmuth, 2015) which could depend on oligomeric quaternary structure.

To conclude, despite many years of combined in vivo, in vitro, and in silico research, many questions about the physiological function of transporter oligomerization and the influence of endogenous and exogenous compounds on oligomerization remain open. We will need novel experimental systems capable of addressing the complexity of the process to further our attempts in understanding SLC6 transporter oligomerization.

## CONFLICTS OF INTEREST

HHS has received honoraria for lectures and consulting from AbbVie, Amgen, AstraZeneca, Astropharma, Bano Healthcare, Chiesi, FOPI, Gebro, IIR, Janssen‐Cilag, Lundbeck, MSD, Novartis, Pfizer, Roche, Sanofi‐Aventis, Shire, Vertex (past 5 years). MEAR has received a consultation fee from Jazz Pharmaceuticals (past 5 years).
